# Closed-Form Solution of the Unit Normal Loss Integral in 2 Dimensions, with Application in Value-of-Information Analysis

**DOI:** 10.1177/0272989X231171166

**Published:** 2023-06-02

**Authors:** Tae Yoon Lee, Paul Gustafson, Mohsen Sadatsafavi, for the Canadian Respiratory Research Network

**Affiliations:** Respiratory Evaluation Sciences Program, Collaboration for Outcomes Research and Evaluation, Faculty of Pharmaceutical Sciences, University of British Columbia, Vancouver, BC, Canada; Department of Statistics, University of British Columbia, Vancouver, BC, Canada; Respiratory Evaluation Sciences Program, Collaboration for Outcomes Research and Evaluation, Faculty of Pharmaceutical Sciences, University of British Columbia, Vancouver, BC, Canada

**Keywords:** decision analysis, risk prediction modeling, cost-effectiveness, uncertainty, value of information

## Abstract

The unit normal loss integral (UNLI) has wide applicability in decision analysis and risk modeling, including as a solution for computation of various value-of-information (VoI) metrics. However, one limitation of the UNLI has been that its closed-form solution is available for only 1 dimension and thus can only be used for comparisons involving 2 strategies (where it is applied to the scalar incremental net benefit). We derived a closed-form solution for the 2-dimensional UNLI by the integration by parts technique. This enables the extension of the UNLI method to 3-comparison problems. We implemented this approach in R as part of the *predtools* package (https://github.com/resplab/predtools/) and verified the accuracy of this implementation via Monte Carlo simulations. A case study based on a 3-arm clinical trial was used as an example for VoI analysis. Methods based on the closed-form solutions for the UNLI can now be extended to 3-decision comparisons, taking a fraction of a second to compute and not being subject to Monte Carlo error.

**Highlights:**

- The unit normal loss integral (UNLI) is widely used in decision analysis and risk modeling, including in the computation of various value-of-information metrics, but its closed-form solution is only applicable to comparisons of 2 strategies.
- We derive a closed-form solution for 2-dimensional UNLI, extending the applicability of the UNLI to 3-strategy comparisons.
- Such closed-form computation takes only a fraction of a second and is free from simulation errors that affect the hitherto available methods.
- In addition to the relevance in 3-strategy model-based and data-driven decision analyses, a particular application is in risk prediction modeling, where the net benefit of a classifier should always be compared with 2 default strategies of treating none and treating all.

The unit normal loss integral (UNLI), first discussed by Raiffa and Schlaifer in the 1960s, has emerged in various ways in decision analysis and risk modeling, including in value-of-information (VoI) analysis.^1^ VoI analysis is a set of concepts and methods rooted in decision theory that quantifies the expected utility loss due to uncertainty associated with decisions.^2^ VoI has been applied in decision analysis across areas including health technology assessment,^3^ environmental risk analysis,^4^ and clinical prediction modeling.^5^ VoI calculations are often carried out using Monte Carlo (MC) simulations, either through repeated sampling of uncertain input parameters in model-based evaluations or via bootstrapping in data-driven analyses.^6^

An overview of UNLI-based methods for VoI computations is provided by Wilson.^7^ The UNLI is closely related to the mean of the truncated normal distribution. The exact definition of the UNLI has varied slightly in different publications.^7–9^ Here we use the following definition. Suppose Y has a normal distribution with mean μ and variance $\sigma^{2}.$ With $\phi \left(y\right)=\frac{e^{\frac{-y^{2}}{2}}\;}{\sqrt{2\pi }}$ and $\Phi \left(y\right)=\int_{-\infty }^{y}\phi \left(t\right)\mathrm{dt}$ denoting, respectively, the probability density and cumulative distribution functions of the standard normal distribution, the UNLI can be defined as

$$\begin{array}{cc} \mathrm{UNLI} & =E\left(\max\left(Y,0\right)\right)=\int_{0}^{\infty }y\;\frac{e^{\frac{-{\left(y-\mu \right)}^{2}}{2\sigma^{2}}}\;}{\sqrt{2\pi }\sigma }\mathrm{dy}\\ & =\int_{0}^{\infty }\frac{y}{\sigma }\;\phi \left(\frac{y-\mu }{\sigma }\right)\mathrm{dy}\;=\mu \left[1-\Phi \left(-\frac{\mu }{\sigma }\right)\right]+\sigma \phi \left(-\frac{\mu }{\sigma }\right). \end{array}$$

A typical application in VoI analysis is the computation of the expected value of perfect information (EVPI).^1,10^ EVPI is the expected gain in net benefit (NB) when uncertainty in the evidence underlying the decision is completely resolved.^7^ When comparing 2 strategies (e.g., use of a new medication versus continuing with standard of care), the outcome of a probabilistic decision analysis can be summarized as a distribution of the incremental NB between the 2 strategies. If this quantity has a normal distribution, then the EVPI can be expressed as a closed-form solution using the 1-dimensional UNLI, as outlined above.^1,10^

This approach for EVPI calculation is applicable to both model-based and data-driven evaluations. The UNLI method has been extended to other VoI metrics, such as expected value of partial perfect information^11^ and expected value of sample information.^12^ Such solutions are computationally feasible and free from MC error, albeit requiring the assumption of normality to hold. However, in many practical decision analyses, there are more than 2 strategies that are compared, making the closed-form UNLI not readily applicable. Approximate methods have been suggested for more than 2 strategies. For example, Jalal et al.^8^ used the UNLI as an approximate solution to multiple comparisons by segmenting the joint probability space of input parameters into adjacent pieces to turn the problem into a sum of 1-dimensional evaluations.

To the best of our knowledge, no closed-form expression for the UNLI has been proposed for higher dimensions. In this work, we derive a closed-form solution for the UNLI for 2 dimensions, enabling the extension of this method to comparisons of 3 strategies. We perform a simulation study to verify the numerical accuracy of our implementation and show its utility in a case study involving EVPI calculation for a data-driven decision analysis based on a 3-arm clinical trial.

## Closed-Form Solution

Suppose we have 3 strategies of interest, with one of them labeled as the reference strategy (the choice of which strategy being designated as the reference has no bearing on the computation). The incremental NBs of the two alternative strategies compared with the reference strategy are denoted by $Y_{1}$ and $Y_{2}$ . Further, suppose that our knowledge of $Y=\left(Y_{1},Y_{2}\right)$ can be expressed as a bivariate normal distribution with mean $\left(\mu_{1},\mu_{2}\right)$ , variance $\left(\sigma_{1}^{2},\sigma_{2}^{2}\right)$ , and correlation coefficient ρ . The expected NB under perfect information is the expectation of max(Y,0) .

Following the derivations provided in the Supplementary Material Section 1, we arrive at the closed-form equation:

$$E\left(\max\left(Y,0\right)\right)=r_{1,2}+q_{1,2}+r_{2,1}+q_{2,1}$$

with each term on the right-hand side defined below. In what follows, 1{condition}=1 if the condition is true and 0 otherwise; $\Phi_{2}\left(x_{1},x_{2},\rho \right)$ is the cumulative density function of the standard bivariate normal with the upper limits and $x_{2}$ and correlation coefficient ρ , and

$$\delta_{i,j}=\;\left\{ \begin{array}{c} 1\;\mathrm{if}\;\sigma_{i}-\rho \sigma_{j}>0\\ -1\;\mathrm{if}\;\sigma_{i}-\rho \sigma_{j}<0 \end{array}\right.$$

$$\begin{array}{cc} & r_{i,j}=\mu_{i}\\ & \left[1\{\left(\sigma_{i}-\rho \sigma_{j}\right)>0\}+\Phi \left(\frac{\rho \sigma_{j}\mu_{i}-\sigma_{i}\mu_{j}}{\sigma_{i}\sigma_{j}\sqrt{\left(1-\rho^{2}\right)}}\right)1\left\{\left(\sigma_{i}-\rho \sigma_{j}\right)=0\right\}\right]-\\ & \Phi \left(\frac{\rho \sigma_{j}\mu_{i}-\sigma_{i}\mu_{j}}{\sigma_{i}\sigma_{j}\sqrt{\left(1-\rho^{2}\right)}}\right)\left(-\sigma_{i}\phi \left(\frac{-\mu_{i}}{\sigma_{i}}\right)+\mu_{i}\Phi \left(\frac{-\mu_{i}}{\sigma_{i}}\right)\right), \end{array}$$

and

$$\begin{array}{cc} & q_{i,j}=1\left\{\left(\sigma_{i}-\rho \sigma_{j}\right)\neq 0\right\}\;\mu_{i}\delta_{i,j}[\Phi \left(\frac{\delta_{i,j}\left(\mu_{j}-\mu_{i}\right)}{\sqrt{\left(\sigma_{i}^{2}+\sigma_{j}^{2}-2\rho \sigma_{i}\sigma_{j}\right)}}\right)\\ & -\Phi_{2}\left(\;\frac{\delta_{i,j}(\mu_{j}-\mu_{i})}{\sqrt{\left(\sigma_{i}^{2}+\sigma_{j}^{2}-2\rho \sigma_{i}\sigma_{j}\right)}},\frac{\delta_{i,j}\left(\rho \sigma_{j}\mu_{i}-\sigma_{i}\mu_{j}\right)}{\sigma_{i}\sigma_{j}\sqrt{\left(1-\rho^{2}\right)}},\;\frac{-\sigma_{j}\sqrt{\left(1-\rho^{2}\right)}}{\sqrt{\left(\sigma_{i}^{2}+\sigma_{j}^{2}-2\rho \sigma_{i}\sigma_{j}\right)}}\right)]\\ & -1\left\{\left(\sigma_{i}-\rho \sigma_{j}\right)\neq 0\right\}\;\delta_{i,j}\sigma_{i}\frac{\left(\sigma_{i}^{2}+\sigma_{j}^{2}-2\rho \sigma_{i}\sigma_{j}\right)}{\sigma_{i}-\rho \sigma_{j}}\phi \left(\frac{\mu_{j}-\mu_{i}}{\left(\sigma_{i}^{2}+\sigma_{j}^{2}-2\rho \sigma_{i}\sigma_{j}\right)}\right)\\ & \left(1-\Phi \left(-\delta_{i,j}\frac{\sigma_{i}^{2}+\sigma_{j}^{2}-2\rho \sigma_{i}\sigma_{j}\;}{\sigma_{i}-\rho \sigma_{j}}\frac{\sigma_{i}\mu_{j}-\rho \sigma_{j}\mu_{i}}{\sigma_{i}\sigma_{j}\sqrt{\left(1-\rho^{2}\right)}}+\delta_{i,j}\frac{\sigma_{j}\sqrt{\left(1-\rho^{2}\right)}}{\left(\sigma_{i}-\rho \sigma_{j}\right)}\;\frac{\mu_{j}-\mu_{i}}{\left(\sigma_{i}^{2}+\sigma_{j}^{2}-2\rho \sigma_{i}\sigma_{j}\right)}\right)\right) \end{array}$$

## Implementation and a Simulation Study

We implemented this method in R (*mu_max_trunc_bvn* function in the *predtools* package - https://github.com/resplab/predtools). To verify the accuracy of our implementation, we conducted a simulation study by comparing it with large-scale MC integrations (*N* = 100,000). We examined 252 permutations of the parameters of the bivariate distributions characterized by a factorial design for the following variables: $\mu_{1},\mu_{2}=\;\left\{-2,\;0,\;2\right\}$ , $\sigma_{1}^{2},\sigma_{2}^{2}=\left\{1,\;3\right\}$ , and ρ={−0.75,−0.50,…,0.50,0.75}. Results show that the difference between the closed-form and MC solutions falls within the range of the MC standard error (Supplementary Material Section 2).

## Case Study

The Optimal Therapy of Chronic Obstructive Pulmonary Disease (COPD) was a parallel-arm clinical trial of 3 inhaler therapies for patients with COPD. In this 3-arm study, 449 patients with COPD were randomized to receive single-inhaler (*n* = 145), double-inhaler (*n* = 156), or triple-inhaler (*n* = 148) therapies.^13^ The trial duration was 12 mo. The study collected data, including monthly cost diaries, and functional scores measured by St. George’s Respiratory Questionnaire at baseline and 4 follow-up visits (at 4, 20, 36, and 52 wk).

The NB calculations in this case study closely follow the methods used in a previously published data-driven economic evaluation of this trial.^14^ The functional scores were converted to EQ5D utilities using validated algorithms.^15^ With the quality-adjusted life years (QALYs) as the health outcome of interest, the single-inhaler strategy dominated the double-inhaler strategy, and the incremental cost-effectiveness ratio of the triple-inhaler therapy versus the single-inhaler therapy was $(4,042−2,678)/[(0.7217−0.7092)QALY] = $243,180/QALY gained (in 2006 Canadian dollars). As such, the single-inhaler therapy was the optimal strategy at the willingness-to-pay value of $50,000/QALY. However, a bootstrap-based probabilistic sensitivity analysis demonstrated uncertainty in the results: the single-inhaler therapy was the optimal strategy in 80% of the bootstraps.

We calculated the EVPI for this evaluation based on such individual-level data from the trial, comparing the bootstrap-based approach^6^ with the proposed UNLI method. At a given willingness-to-pay value, the NB was calculated as QALY multiplied by willingness-to-pay minus total costs for each patient. Then, for each arm, we calculated the average NB. Seven percent of costs and utility values were missing. Similar to the approach used in the original evaluation, we imputed the missing values using multiple imputation by chained equation with predictive mean matching.^16^ For the bootstrap-based approach, imputation was embedded within bootstrapping, such that each iteration of the MC simulation involved one imputation and generation of a single bootstrapped sample. This was repeated 1,000 times. For the UNLI method, we generated 10 imputed data sets and pooled the mean and covariance matrix estimates of incremental NBs using the Rubin’s rule.^17^ Next, taking the single-inhaler therapy as the reference, the incremental NBs of double- and triple-inhaler therapies were parameterized as a bivariate normal distribution. For example, at a willingness-to-pay of $50,000/QALY, the pooled parameter values were $\mu_{1}=-4,734$ , $\mu_{2}=-2,668$ , $\sigma_{1}=4,678$ , $\sigma_{2}=4,645$ , and ρ = 0.50, where subscripts 1 and 2 indicate the incremental NB of double- and triple-inhaler therapies, respectively, compared with single-inhaler therapy.

**Figure 1:**
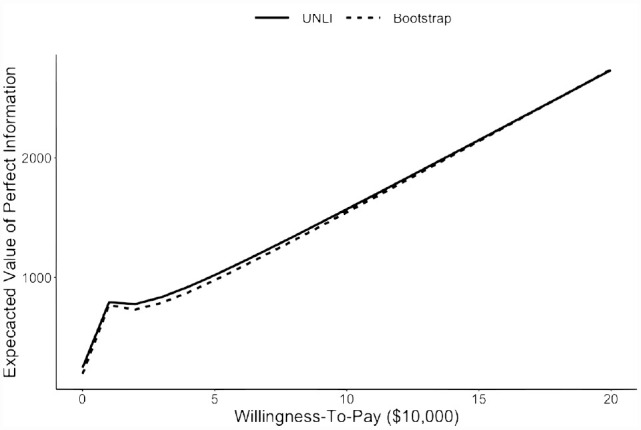
Expected value of perfect information based on the closed-form unit normal loss integral (UNLI) method (solid) and bootstrap method (dashed).

EVPI values were similar between the closed-form UNLI and bootstrap methods with the mean relative absolute error of 3% (Figure 1). At a willingness-to-pay of $50,000/QALY, the EVPI values were $1,019 for the closed-form UNLI method and $976 (MC standard error: 61) for the bootstrap method. At a willingness-to-pay of $100,000/QALY, the corresponding values were $1,570 and $1,543 (MC standard error: 107).

## Discussion

We have proposed a closed-form solution for the 2-dimensional version of the UNLI, enabling the extension of existing methods based on this integral to 3-strategy decision problems, with particular application in VoI analysis. Provided the assumption of the bivariate normality of the incremental NBs is ascertained, the UNLI method provides a solution that takes a fraction of a second to compute and is not subject to the MC error inherent in simulation-based methods. The R code for this method is provided in the *predtools* package, along with a tutorial (https://resplab.github.io/predtools/articles/UNLI2D.html).

In a case study, we compared the UNLI method with the conventional bootstrap-based approach for EVPI computations for a data-driven economic evaluation based on data from a 3-arm clinical trial. We found that the results from the bootstrap-based approach and the UNLI-based approach were very close. As the UNLI-based approach is computationally faster and free from MC error, in similar contexts, presenting the UNLI-based results in VoI analysis would help with the reproducibility of results.

A key condition for the validity of UNLI-based computations is the normality assumption (bivariate normality in the 2-dimensional case). If this condition is violated, the UNLI-based approach could produce misleading results (in which case, more flexible methods should be considered). In data-driven evaluations, provided that the sample size is sufficient, the central limit theorem provides a general justification for the normality assumption with sufficient sample sizes.^7^ For model-based evaluation, the appropriateness of the normality assumption must be scrutinized, as has been demonstrated in the previous applications of the 1-dimensional UNLI method.^18^

In addition to decision-analytic problems, the 2-dimensional UNLI can be particularly relevant for the recently proposed VoI analysis in risk prediction modeling.^5^ The NB calculation for a risk prediction model is naturally a 3-decision problem, as a risk prediction model must be always compared with at least the 2 default strategies of “treating none” and “treating all.” Here again, the central limit theorem provides justification for the assumption of (bivariate) normality, for example, for the distribution of incremental NB of “treating all” and “model-based treatment” with “treating none” as the reference strategy.^19^ The Wald-type method proposed by Marsh et al. can be used to derive the moments of the bivariate normal distribution, enabling UNLI-based VoI analysis.^20,21^

In this work, we focused on EVPI calculation to demonstrate the applicability of the 2-dimensional UNLI method. The 1-dimensional UNLI method has been expanded to other VoI metrics, including the expected value of partial perfect information and the expected value of sample information.^7,8^ Those algorithms can be feasibly modified to accommodate decision analyses with 3 strategies using the 2-dimensional UNLI method.

A natural question is whether the UNLI method can be extended to higher dimensions. We believe that there is no closed-form solution for the general *m*-dimensional case. The *m*-dimensional UNLI is closely related to the expression for the maximum of *m*-variate normal random variables. Arellano-Valle and Genton showed (corollary 4) that for *m* > 2, the probability density function of the maximum of *m*-variate normal random variables is no longer composed of univariate normal distribution functions.^22^ Under the assumption of independence, DasGupta provided a closed-form solution for the expectation of 3 normal random variables and asserted that such a solution is unlikely to exist for *m* > 3.^23^ Thus, it is not surprising that for a general setting, only bounds on the expectation, or infinite series expansions, rather than a closed-form solution, have been computed in the literature.^24^

## Supplemental Material

sj-docx-1-mdm-10.1177_0272989X231171166 – Supplemental material for Closed-Form Solution of the Unit Normal Loss Integral in 2 Dimensions, with Application in Value-of-Information Analysis Click here for additional data file. Supplemental material, sj-docx-1-mdm-10.1177_0272989X231171166 for Closed-Form Solution of the Unit Normal Loss Integral in 2 Dimensions, with Application in Value-of-Information Analysis by Tae Yoon Lee, Paul Gustafson and Mohsen Sadatsafavi in Medical Decision Making
