# Supplementary material for: Closed-Form Solution of the Unit Normal Loss Integral in 2 Dimensions, with Application in Value-of-Information Analysis
Source: Med Decis Making. 2023 Jun 2;43(5):621–6. doi: 10.1177/0272989X231171166 (PMC10336700; doi:10.1177/0272989X231171166)
Supplement: sj-docx-1-mdm-10.1177_0272989X231171166 – Supplemental material for Closed-Form Solution of the Unit Normal Loss Integral in 2 Dimensions, with Application in Value-of-Information Analysis [file sj-docx-1-mdm-10.1177_0272989X231171166.docx]

**Supplementary Materials**

**1. Derivation of the closed-form solution for two-dimension UNLI**

Suppose we have two strategies of interest in comparison to a reference strategy, with corresponding two scalar incremental net benefits, denoted by $Y_{1}$ and $Y_{2}$. Further suppose that $Y=\left( Y_{1},Y_{2} \right)$ follow a bivariate normal distribution with mean $\left( \mu_{1},\mu_{2} \right)$, variance $\left( \sigma_{1}^{2},\sigma_{2}^{2} \right)$, and correlation coefficient $\rho$. Our target quantity is the expectation of $max\left( Y,0 \right)$:

$$E\left( \max\left( Y,0 \right) \right)=E\left( Y\boldsymbol{1}\{Y>0\}+0\boldsymbol{1}\{Y\leq0\} \right)$$

$$=E\left( Y\boldsymbol{1}\{Y>0\} \right)$$

$$=\int_{-\infty}^{\infty} yf_{Y}\left( y \right)\boldsymbol{1}\{Y>0\}dy$$

$$=\int_{-\infty}^{\infty} yf_{Y_{1}}\left( -y \right)\boldsymbol{1}\{Y>0\}dy+\int_{-\infty}^{\infty} yf_{Y_{2}}\left( -y \right)\boldsymbol{1}\{Y>0\}dy,$$

where $f_{Y}\left( y \right)$ is the probability density function of Y, $\boldsymbol{1}\left( \mathrm{condition} \right)=1$if the condition is true and 0 otherwise, and the last equation follows from the decomposition of the bivariate normal probability density function,[1] with $\phi$ and $\Phi$denoting the probability density and cumulative distribution functions of the standard normal distribution:

$f_{X_{i}}\left( x_{i} \right)=\frac{1}{\sigma_{i}}\phi\left( \frac{x_{i}+\mu_{i}}{\sigma_{i}} \right)\Phi\left( \frac{\rho\left( x_{i}+\mu_{i} \right)}{\sigma_{i}\sqrt{\left( 1-\rho^{2} \right)}}-\frac{x_{i}+\mu_{j}}{\sigma_{j}\sqrt{\left( 1-\rho^{2} \right)}} \right), i \neq j, i,j=1,2$.

We compute the first integral of $E\left( \max\left( Y,0 \right) \right)$ and later derive the expression of the second integral by symmetry:

$$\int_{-\infty}^{\infty} yf_{Y_{1}}\left( -y \right)1\{Y>0\}dy=\int_{0}^{\infty} y\frac{1}{\sigma_{1}}\phi\left( \frac{y-\mu_{1}}{\sigma_{1}} \right)\Phi\left( \frac{\sigma_{1}\left( y-\mu_{2} \right)-\sigma_{2}\rho\left( y-\mu_{1} \right)}{\sigma_{1}\sigma_{2}\sqrt{\left( 1-\rho^{2} \right)}} \right)dy.$$

We apply the integration by parts technique to solve this integral in two components, with

$$u_{1,2}=\Phi\left( \frac{\sigma_{1}\left( y-\mu_{2} \right)-\sigma_{2}\rho\left( y-\mu_{1} \right)}{\sigma_{1}\sigma_{2}\sqrt{\left( 1-\rho^{2} \right)}} \right)=\Phi\left( \frac{y\left( \sigma_{1}-\rho\sigma_{2} \right)-\sigma_{1}\mu_{2}+\rho\sigma_{2}\mu_{1}}{\sigma_{1}\sigma_{2}\sqrt{\left( 1-\rho^{2} \right)}} \right)$$

and

$dv_{1,2}=y\frac{1}{\sigma_{1}}\phi\left( \frac{\left( y-\mu_{1} \right)}{\sigma_{1}} \right)dy$.

Then we have

$$du_{1,2}=\frac{\left( \sigma_{1}-\rho\sigma_{2} \right)}{\sigma_{1}\sigma_{2}\sqrt{\left( 1-\rho^{2} \right)}}\phi\left( \frac{y\left( \sigma_{1}-\rho\sigma_{2} \right)-\sigma_{1}\mu_{2}+\rho\sigma_{2}\mu_{1}}{\sigma_{1}\sigma_{2}\sqrt{\left( 1-\rho^{2} \right)}} \right)dy$$

and

$v_{1,2}=\int_{0}^{\infty} y\frac{1}{\sigma_{1}}\phi\left( \frac{\left( y-\mu_{1} \right)}{\sigma_{1}} \right)dy=\mu_{1}\Phi\left( \frac{y-\mu_{1}}{\sigma_{1}} \right)-\sigma_{1}\phi\left( \frac{y-\mu_{1}}{\sigma_{1}} \right)$.

The first component $r_{1,2}$ is straightforward:

$r_{1,2}:=[u_{1,2}v_{1,2}\left. \right|_{0}^{\infty}=\mu_{1}\left[ \boldsymbol{1}\{\left( \sigma_{1}-\rho\sigma_{2} \right)>0\}+\Phi\left( \frac{\rho\sigma_{2}\mu_{1}-\sigma_{1}\mu_{2}}{\sigma_{1}\sigma_{2}\sqrt{\left( 1-\rho^{2} \right)}} \right)\boldsymbol{1}\{\left( \sigma_{1}-\rho\sigma_{2} \right)=0\} \right]-\Phi\left( \frac{\rho\sigma_{2}\mu_{1}-\sigma_{1}\mu_{2}}{\sigma_{1}\sigma_{2}\sqrt{\left( 1-\rho^{2} \right)}} \right)\left( -\sigma_{1}\phi\left( \frac{-\mu_{1}}{\sigma_{1}} \right)+\mu_{1}\Phi\left( \frac{-\mu_{1}}{\sigma_{1}} \right) \right)$.

We compute term-by-term for the second component $q_{1,2}$with $\alpha_{1,2}=\sigma_{1}\mu_{2}-\rho\sigma_{2}\mu_{1}$ and $\beta=\sigma_{1}\sigma_{2}\sqrt{\left( 1-\rho^{2} \right)}$:

$$q_{1,2}:=\int_{0}^{\infty} v_{1,2}du_{1,2}=\frac{\left( \sigma_{1}-\rho\sigma_{2} \right)}{\beta}\int_{0}^{\infty} \left( \mu_{1}\Phi\left( \frac{y-\mu_{1}}{\sigma_{1}} \right)-\sigma_{1}\phi\left( \frac{y-\mu_{1}}{\sigma_{1}} \right) \right)\phi\left( \frac{\left( \sigma_{1}-\rho\sigma_{2} \right)y-\alpha_{1,2}}{\beta} \right)dy.$$

Let $T_{1}^{1,2}$ and $T_{2}^{1,2}$be the decomposition of $q_{1,2}$:

$$T_{1}^{1,2}=\frac{\mu_{1}\left( \sigma_{1}-\rho\sigma_{2} \right)}{\beta}\int_{0}^{\infty} \Phi\left( \frac{y-\mu_{1}}{\sigma_{1}} \right)\phi\left( \frac{\left( \sigma_{1}-\rho\sigma_{2} \right)y-\alpha_{1,2}}{\beta} \right)dy$$

and

$$T_{2}^{1,2}=\frac{-\sigma_{1}\left( \sigma_{1}-\rho\sigma_{2} \right)}{\beta}\int_{0}^{\infty} \phi\left( \frac{y-\mu_{1}}{\sigma_{1}} \right)\phi\left( \frac{\left( \sigma_{1}-\rho\sigma_{2} \right)y-\alpha_{1,2}}{\beta} \right)dy.$$

Let $\Phi_{2}\left( w_{1},w_{2},\rho\right)$ be the cumulative density of the standard bivariate normal with the upper limits $w_{1},w_{2}$ and correlation coefficient $\rho.$

We use the table of integrals by Owen,[2] and we have two cases depending on $\left( \sigma_{1}-\rho\sigma_{2} \right).$

Case I) If $\sigma_{1}-\rho\sigma_{2}=0,$then $q_{1,2}=0.$

Case II) $\sigma_{1}-\rho\sigma_{2} \neq0.$Let $\delta_{1,2}= sgn\left( \sigma_{1}-\rho\sigma_{2} \right) = \left\{ \begin{aligned} 1 if \sigma_{1}-\rho\sigma_{2}>0 \\ -1 if \sigma_{1}-\rho\sigma_{2}<0 \end{aligned} \right.$.

Then $\left( \sigma_{1}-\rho\sigma_{2} \right) = \delta_{1,2}\left| \sigma_{1}-\rho\sigma_{2} \right|,$ and note that $\delta_{1,2}=\delta_{1,2}$and ${\delta_{1,2}}^{2}=1.$

$$T_{1}^{1,2}=\frac{\mu_{1}\left( \sigma_{1}-\rho\sigma_{2} \right)}{\beta}\int_{0}^{\infty} \Phi\left( \frac{y-\mu_{1}}{\sigma_{1}} \right)\phi\left( \frac{\left( \sigma_{1}-\rho\sigma_{2} \right) y-\alpha_{1,2}}{\beta} \right)dy$$

$\text{use change of variable with x=}\delta_{1,2}\text{(}\left( \sigma_{1}-\rho\sigma_{2} \right)\text{y}-\alpha_{1,2}\text{)}\text{/}\text{β}$= $\text{(}\left| \text{σ}_{\text{1}}\text{-ρ}\text{σ}_{\text{2}} \right|\text{y}-\delta_{1,2}\alpha_{1,2}\text{)}\text{/}\text{β}$

let $\alpha'= \delta_{1,2}\alpha_{1,2}\text{/}\left| \text{σ}_{\text{1}}\text{-ρ}\text{σ}_{\text{2}} \right|$and $\beta'= \text{β/}\left| \text{σ}_{\text{1}}\text{-ρ}\text{σ}_{\text{2}} \right| (>0)$

$$=\frac{\mu_{1}\delta_{1,2}\left| \sigma_{1}-\rho\sigma_{2} \right|}{\beta}\int_{-\delta_{1,2}\alpha_{1,2}/\beta}^{\infty} \phi\left( \delta_{1,2}x \right)\Phi\left( \frac{\beta'x+\alpha'-\mu_{1}}{\sigma_{1}} \right)\left( \frac{\beta}{\left| \text{σ}_{\text{1}}\text{-ρ}\text{σ}_{\text{2}} \right|}dx \right)$$

$$\text{let }a=\left( \mu_{1}-\alpha' \right)/\beta' \text{and }b=\sigma_{1}/\beta'$$

note $\phi\left( \delta_{1,2}x \right)= \phi\left( x \right)$ by symmetry of $\phi(.)$

$$={\delta_{1,2}\mu}_{1}\int_{-\delta_{1,2}\alpha_{1,2}/\beta}^{\infty} \phi\left( x \right)\Phi\left( \left( x-a \right)/b \right)dx$$

$$={\delta_{1,2}\mu}_{1}\left[ \int_{-\infty}^{\infty} \phi\left( x \right)\Phi\left( \left( x-a \right)/b \right)dx-\int_{-\infty}^{-\delta_{1,2}\alpha_{1,2}/\beta} \phi\left( x \right)\Phi\left( \left( x-a \right)/b \right)dx \right]$$

use the table of integral

$$={\delta_{1,2}\mu}_{1}\left[ \Phi\left( \left( -a/b \right)/\left( 1+\left( 1/b \right)^{2} \right)^{0.5} \right)-\Phi_{2}\left( -a/\sqrt{\left( 1+b^{2} \right)},-\delta_{1,2}\alpha_{1,2}/\beta,-1/\sqrt{\left( 1+b^{2} \right)} \right) \right]$$

Note that

$$\left( -a/b \right)/\left( 1+\left( 1/b \right)^{2} \right)^{0.5}=\left( -a/b \right)/\left( \left( b^{2}+1 \right)/b^{2} \right)^{0.5}=-a/\sqrt{1+b^{2}}$$

$$1+b^{2}=1+\left( \frac{\sigma_{1}}{\beta^{'}} \right)^{2}=1+\left( \frac{\sigma_{1}\left| \sigma_{1}-\rho\sigma_{2} \right|}{\sigma_{1}\sigma_{2}\sqrt{\left( 1-\rho^{2} \right)}} \right)^{2}=\frac{\sigma_{2}^{2}\left( 1-\rho^{2} \right)+\left( \sigma_{1}-\rho\sigma_{2} \right)^{2}}{\sigma_{2}^{2}\left( 1-\rho^{2} \right)}=\frac{\sigma_{2}^{2}-\rho^{2}\sigma_{2}^{2}+\sigma_{1}^{2}+\rho^{2}\sigma_{2}^{2}-2{\rho\sigma}_{1}\sigma_{2}}{\sigma_{2}^{2}\left( 1-\rho^{2} \right)}=\frac{\sigma_{2}^{2}+\sigma_{1}^{2}-2{\rho\sigma}_{1}\sigma_{2}}{\sigma_{2}^{2}\left( 1-\rho^{2} \right)}$$

$$a=\frac{\mu_{1}-\alpha^{'}}{\beta^{'}} =\frac{\mu_{1}\left| \text{σ}_{\text{1}}\text{-ρ}\text{σ}_{\text{2}} \right|-\delta_{1,2}\alpha_{1,2}}{\beta} = \frac{\mu_{1}\left( \sigma_{1}-\rho\sigma_{2} \right)-\alpha_{1,2}}{\delta_{1,2}\beta}$$

$$=\frac{\mu_{1}\sigma_{1}-\rho\sigma_{2}\mu_{1}-\sigma_{1}\mu_{2}+\rho\sigma_{2}\mu_{1}}{\delta_{1,2}\sigma_{1}\sigma_{2}\sqrt{\left( 1-\rho^{2} \right)}} =\frac{\delta_{1,2}(\mu_{1}-\mu_{2})}{\sigma_{2}\sqrt{\left( 1-\rho^{2} \right)}}$$

$$-a/\sqrt{1+b^{2}}=\frac{{\delta_{1,2}(\mu}_{2}-\mu_{1})}{\sigma_{2}\sqrt{\left( 1-\rho^{2} \right)}}\frac{\sqrt{\sigma_{2}^{2}\left( 1-\rho^{2} \right)}}{\sqrt{\sigma_{2}^{2}+\sigma_{1}^{2}-2{\rho\sigma}_{1}\sigma_{2}}}=\frac{\delta_{1,2}(\mu_{2}-\mu_{1})}{\sqrt{\sigma_{2}^{2}+\sigma_{1}^{2}-2{\rho\sigma}_{1}\sigma_{2}}}$$

Then in terms of the original parameters,

$${T_{1}^{1,2}=\delta_{1,2}\mu}_{1}\left[ \Phi\left( \frac{\delta_{1,2}(\mu_{2}-\mu_{1})}{\sqrt{\left( \sigma_{1}^{2}+\sigma_{2}^{2}-2\rho\sigma_{1}\sigma_{2} \right)}} \right)-\Phi_{2}\left( \frac{{\delta_{1,2}(\mu}_{2}-\mu_{1})}{\sqrt{\left( \sigma_{1}^{2}+\sigma_{2}^{2}-2\rho\sigma_{1}\sigma_{2} \right)}},\frac{\delta_{1,2}(\rho\sigma_{2}\mu_{1}-\sigma_{1}\mu_{2})}{\sigma_{1}\sigma_{2}\sqrt{\left( 1-\rho^{2} \right)}}, \frac{-\sigma_{2}\sqrt{\left( 1-\rho^{2} \right)}}{\sqrt{\left( \sigma_{1}^{2}+\sigma_{2}^{2}-2\rho\sigma_{1}\sigma_{2} \right)}} \right) \right]$$

Now we turn to calculation of $T_{2}^{1,2}$:

$$T_{2}^{1,2}=\frac{-\sigma_{1}\left( \sigma_{1}-\rho\sigma_{2} \right)}{\beta}\int_{0}^{\infty} \phi\left( \frac{y-\mu_{1}}{\sigma_{1}} \right)\phi\left( \frac{\left( \sigma_{1}-\rho\sigma_{2} \right)y-\alpha_{1,2}}{\beta} \right)dy$$

$$\text{use change of variable }x=\delta_{1,2}\left( \left( \sigma_{1}-\rho\sigma_{2} \right)y-\alpha_{1,2} \right)/\beta=\left( \left| \text{σ}_{\text{1}}\text{-ρ}\text{σ}_{\text{2}} \right|y-\delta_{1,2}\alpha_{1,2} \right)/\beta$$

let $\alpha'= \delta_{1,2}\alpha_{1,2}\text{/}\left| \text{σ}_{\text{1}}\text{-ρ}\text{σ}_{\text{2}} \right|$and $\beta'= \text{β/}\left| \text{σ}_{\text{1}}\text{-ρ}\text{σ}_{\text{2}} \right|$

$$=\frac{-\sigma_{1}\delta_{1,2}\left| \text{σ}_{\text{1}}\text{-ρ}\text{σ}_{\text{2}} \right|}{\beta}\int_{-\delta_{1,2}\alpha_{1,2}/\beta}^{\infty} \phi\left( \frac{x\beta^{'}+\alpha^{'}-\mu_{1}}{\sigma_{1}} \right)\phi\left( \delta_{1,2}x \right)\left( \frac{\beta}{\left| \text{σ}_{\text{1}}\text{-ρ}\text{σ}_{\text{2}} \right|}dx \right)$$

$$=-{\delta\sigma}_{1}\int_{-\delta_{1,2}\alpha_{1,2}/\beta}^{\infty} \phi\left( \frac{x\beta'+\alpha'-\mu_{1}}{\sigma_{1}} \right)\phi\left( x \right) dx$$

$$\text{let }a=\left( \alpha'-\mu_{1} \right)/\sigma_{1},b=\beta'/\sigma_{1},t=\sqrt{1+b^{2}}$$

$$=-\delta_{1,2}\sigma_{1}\int_{-\delta_{1,2}\alpha_{1,2}/\beta}^{\infty} \phi\left( a+bx \right)\phi\left( x \right)dx$$

use the table of integral

$$=-{\delta_{1,2}\sigma}_{1} [\frac{1}{t}\phi\left( a/t \right)\Phi\left( tx+ab/t \right)\left. \right|_{-\delta_{1,2}\alpha_{1,2}/\beta}^{\infty}$$

$$=\frac{-{\delta_{1,2}\sigma}_{1}}{t}\phi\left( a/t \right)\left( 1-\Phi\left( -\delta_{1,2}t\alpha_{1,2}/\beta+ab/t \right) \right)$$

Note that

$$a =\frac{\left( \alpha^{'}-\mu_{1} \right)}{\sigma_{1}}=\frac{\delta_{1,2}\alpha_{1,2}-\mu_{1}\left| \text{σ}_{\text{1}}\text{-ρ}\text{σ}_{\text{2}} \right|}{\sigma_{1}\left| \text{σ}_{\text{1}}\text{-ρ}\text{σ}_{\text{2}} \right|}=\frac{\alpha_{1,2}-\mu_{1}\left( \sigma_{1}-\rho\sigma_{2} \right)}{\sigma_{1}\left( \sigma_{1}-\rho\sigma_{2} \right)}=\frac{\sigma_{1}\mu_{2}-\rho\sigma_{2}\mu_{1}-\mu_{1}\sigma_{1}+\rho\sigma_{2}\mu_{1}}{\sigma_{1}\left( \sigma_{1}-\rho\sigma_{2} \right)}$$

$$= \frac{\sigma_{1}\mu_{2}-\mu_{1}\sigma_{1}}{\sigma_{1}(\sigma_{1}-\rho\sigma_{2})}= \frac{\mu_{2}-\mu_{1}}{(\sigma_{1}-\rho\sigma_{2})}$$

$$t=\sqrt{1+b^{2}}=\frac{\sigma_{1}^{2}+\sigma_{2}^{2}-2{\rho\sigma}_{1}\sigma_{2}}{\sigma_{1}-\rho\sigma_{2}}$$

$$\frac{a}{t}=\frac{\mu_{2}-\mu_{1}}{\left( \sigma_{1}^{2}+\sigma_{2}^{2}-2{\rho\sigma}_{1}\sigma_{2} \right)}$$

Then in terms of the original parameters,

$$T_{2}^{1,2}=-{\delta_{1,2}\sigma}_{1}\frac{\left( \sigma_{1}^{2}+\sigma_{2}^{2}-2{\rho\sigma}_{1}\sigma_{2} \right)}{\sigma_{1}-\rho\sigma_{2}}\phi\left( \frac{\mu_{2}-\mu_{1}}{\left( \sigma_{1}^{2}+\sigma_{2}^{2}-2{\rho\sigma}_{1}\sigma_{2} \right)} \right)$$

$$\left( 1-\Phi\left( -\delta_{1,2}\frac{\sigma_{1}^{2}+\sigma_{2}^{2}-2{\rho\sigma}_{1}\sigma_{2}}{\sigma_{1}-\rho\sigma_{2}}\frac{\sigma_{1}\mu_{2}-\rho\sigma_{2}\mu_{1}}{\sigma_{1}\sigma_{2}\sqrt{\left( 1-\rho^{2} \right)}}+\delta_{1,2}\frac{\sigma_{2}\sqrt{\left( 1-\rho^{2} \right)}}{\left( \sigma_{1}-\rho\sigma_{2} \right)} \frac{\mu_{2}-\mu_{1}}{\left( \sigma_{1}^{2}+\sigma_{2}^{2}-2{\rho\sigma}_{1}\sigma_{2} \right)} \right) \right)$$

Collecting all the components,

$$q_{1,2}= \boldsymbol{1}\{\left( \sigma_{1}-\rho\sigma_{2} \right)\neq0\}\mu_{1}\delta_{1,2}\left[ \Phi\left( \frac{\delta_{1,2}(\mu_{2}-\mu_{1})}{\sqrt{\left( \sigma_{1}^{2}+\sigma_{2}^{2}-2\rho\sigma_{1}\sigma_{2} \right)}} \right)-\Phi_{2}\left( \frac{{\delta_{1,2}(\mu}_{2}-\mu_{1})}{\sqrt{\left( \sigma_{1}^{2}+\sigma_{2}^{2}-2\rho\sigma_{1}\sigma_{2} \right)}},\frac{\delta_{1,2}(\rho\sigma_{2}\mu_{1}-\sigma_{1}\mu_{2})}{\sigma_{1}\sigma_{2}\sqrt{\left( 1-\rho^{2} \right)}}, \frac{-\sigma_{2}\sqrt{\left( 1-\rho^{2} \right)}}{\sqrt{\left( \sigma_{1}^{2}+\sigma_{2}^{2}-2\rho\sigma_{1}\sigma_{2} \right)}} \right) \right]$$

$$- \boldsymbol{1}\{\left( \sigma_{1}-\rho\sigma_{2} \right)\neq0\}{\delta_{1,2}\sigma}_{1}\frac{\left( \sigma_{1}^{2}+\sigma_{2}^{2}-2{\rho\sigma}_{1}\sigma_{2} \right)}{\sigma_{1}-\rho\sigma_{2}}\phi\left( \frac{\mu_{2}-\mu_{1}}{\left( \sigma_{1}^{2}+\sigma_{2}^{2}-2{\rho\sigma}_{1}\sigma_{2} \right)} \right)$$

$$\left( 1-\Phi\left( -\delta_{1,2}\frac{\sigma_{1}^{2}+\sigma_{2}^{2}-2{\rho\sigma}_{1}\sigma_{2}}{\sigma_{1}-\rho\sigma_{2}}\frac{\sigma_{1}\mu_{2}-\rho\sigma_{2}\mu_{1}}{\sigma_{1}\sigma_{2}\sqrt{\left( 1-\rho^{2} \right)}}+\delta_{1,2}\frac{\sigma_{2}\sqrt{\left( 1-\rho^{2} \right)}}{\left( \sigma_{1}-\rho\sigma_{2} \right)} \frac{\mu_{2}-\mu_{1}}{\left( \sigma_{1}^{2}+\sigma_{2}^{2}-2{\rho\sigma}_{1}\sigma_{2} \right)} \right) \right)$$

By symmetry, we can compute the second integral of $E\left( \max\left( Y,0 \right) \right)$ as ${r_{2,1}+q}_{2,1}.$

Thus,

$$E\left( \max\left( Y,0 \right) \right)={{{r_{1,2}+q}_{1,2}+r}_{2,1}+q}_{2,1}.$$

**2. Simulation results**

**Table 1.** Comparison of the closed-form unit normal loss integral (UNLI) and Monte Carlo (MC; N=100,000) solutions for 252 different bivariate distributions.

| $\boldsymbol{\mu}_{\boldsymbol{1}}$ | $\boldsymbol{\mu}_{\boldsymbol{2}}$ | $\boldsymbol{\sigma}_{\boldsymbol{1}}^{\boldsymbol{2}}$ | $\boldsymbol{\sigma}_{\boldsymbol{2}}^{\boldsymbol{2}}$ | $\boldsymbol{\rho}$ | **UNLI** | **MC** | **MC standard error** | $\boldsymbol{\mu}_{\boldsymbol{1}}$ | $\boldsymbol{\mu}_{\boldsymbol{2}}$ | $\boldsymbol{\sigma}_{\boldsymbol{1}}^{\boldsymbol{2}}$ | $\boldsymbol{\sigma}_{\boldsymbol{2}}^{\boldsymbol{2}}$ | $\boldsymbol{\rho}$ | **UNLI** | **MC** | **MC standard error** |
| --- | --- | --- | --- | --- | --- | --- | --- | --- | --- | --- | --- | --- | --- | --- | --- |
| -2 | -2 | 1 | 1 | -0.75 | 0.0170 | 0.0172 | 0.0003 | 0 | 2 | 3 | 1 | 0.00 | 2.1702 | 2.1746 | 0.0031 |
| 0 | -2 | 1 | 1 | -0.75 | 0.4074 | 0.4063 | 0.0018 | 2 | 2 | 3 | 1 | 0.00 | 2.7987 | 2.8024 | 0.0037 |
| 2 | -2 | 1 | 1 | -0.75 | 2.0139 | 2.0101 | 0.0031 | -2 | -2 | 1 | 3 | 0.00 | 0.1143 | 0.1157 | 0.0012 |
| -2 | 0 | 1 | 1 | -0.75 | 0.4074 | 0.4061 | 0.0018 | 0 | -2 | 1 | 3 | 0.00 | 0.4766 | 0.4803 | 0.0020 |
| 0 | 0 | 1 | 1 | -0.75 | 0.7721 | 0.7684 | 0.0020 | 2 | -2 | 1 | 3 | 0.00 | 2.0240 | 2.0275 | 0.0031 |
| 2 | 0 | 1 | 1 | -0.75 | 2.1364 | 2.1336 | 0.0027 | -2 | 0 | 1 | 3 | 0.00 | 0.6959 | 0.6963 | 0.0032 |
| -2 | 2 | 1 | 1 | -0.75 | 2.0139 | 2.0149 | 0.0031 | 0 | 0 | 1 | 3 | 0.00 | 0.9439 | 0.9433 | 0.0031 |
| 0 | 2 | 1 | 1 | -0.75 | 2.1364 | 2.1397 | 0.0027 | 2 | 0 | 1 | 3 | 0.00 | 2.1702 | 2.1708 | 0.0031 |
| 2 | 2 | 1 | 1 | -0.75 | 2.7464 | 2.7441 | 0.0021 | -2 | 2 | 1 | 3 | 0.00 | 2.1080 | 2.1103 | 0.0049 |
| -2 | -2 | 3 | 1 | -0.75 | 0.1150 | 0.1164 | 0.0012 | 0 | 2 | 1 | 3 | 0.00 | 2.1955 | 2.1916 | 0.0047 |
| 0 | -2 | 3 | 1 | -0.75 | 0.6995 | 0.6971 | 0.0032 | 2 | 2 | 1 | 3 | 0.00 | 2.7987 | 2.8019 | 0.0037 |
| 2 | -2 | 3 | 1 | -0.75 | 2.1143 | 2.1035 | 0.0049 | -2 | -2 | 3 | 3 | 0.00 | 0.2059 | 0.2067 | 0.0016 |
| -2 | 0 | 3 | 1 | -0.75 | 0.5046 | 0.5029 | 0.0020 | 0 | -2 | 3 | 3 | 0.00 | 0.7606 | 0.7545 | 0.0032 |
| 0 | 0 | 3 | 1 | -0.75 | 1.0573 | 1.0539 | 0.0029 | 2 | -2 | 3 | 3 | 0.00 | 2.1329 | 2.1295 | 0.0049 |
| 2 | 0 | 3 | 1 | -0.75 | 2.3215 | 2.3223 | 0.0043 | -2 | 0 | 3 | 3 | 0.00 | 0.7606 | 0.7603 | 0.0032 |
| -2 | 2 | 3 | 1 | -0.75 | 2.0668 | 2.0647 | 0.0029 | 0 | 0 | 3 | 3 | 0.00 | 1.1796 | 1.1718 | 0.0036 |
| 0 | 2 | 3 | 1 | -0.75 | 2.3206 | 2.3221 | 0.0026 | 2 | 0 | 3 | 3 | 0.00 | 2.3229 | 2.3182 | 0.0046 |
| 2 | 2 | 3 | 1 | -0.75 | 3.0248 | 3.0313 | 0.0031 | -2 | 2 | 3 | 3 | 0.00 | 2.1329 | 2.1355 | 0.0048 |
| -2 | -2 | 1 | 3 | -0.75 | 0.1150 | 0.1145 | 0.0012 | 0 | 2 | 3 | 3 | 0.00 | 2.3229 | 2.3017 | 0.0046 |
| 0 | -2 | 1 | 3 | -0.75 | 0.5046 | 0.5038 | 0.0020 | 2 | 2 | 3 | 3 | 0.00 | 2.9844 | 2.9860 | 0.0045 |
| 2 | -2 | 1 | 3 | -0.75 | 2.0668 | 2.0632 | 0.0029 | -2 | -2 | 1 | 1 | 0.25 | 0.0166 | 0.0166 | 0.0003 |
| -2 | 0 | 1 | 3 | -0.75 | 0.6995 | 0.7033 | 0.0032 | 0 | -2 | 1 | 1 | 0.25 | 0.4021 | 0.4035 | 0.0019 |
| 0 | 0 | 1 | 3 | -0.75 | 1.0573 | 1.0609 | 0.0029 | 2 | -2 | 1 | 1 | 0.25 | 2.0086 | 2.0094 | 0.0031 |
| 2 | 0 | 1 | 3 | -0.75 | 2.3206 | 2.3196 | 0.0026 | -2 | 0 | 1 | 1 | 0.25 | 0.4021 | 0.4024 | 0.0018 |
| -2 | 2 | 1 | 3 | -0.75 | 2.1143 | 2.1125 | 0.0049 | 0 | 0 | 1 | 1 | 0.25 | 0.6432 | 0.6429 | 0.0021 |
| 0 | 2 | 1 | 3 | -0.75 | 2.3215 | 2.3244 | 0.0042 | 2 | 0 | 1 | 1 | 0.25 | 2.0317 | 2.0304 | 0.0030 |
| 2 | 2 | 1 | 3 | -0.75 | 3.0248 | 3.0310 | 0.0031 | -2 | 2 | 1 | 1 | 0.25 | 2.0086 | 2.0111 | 0.0031 |
| -2 | -2 | 3 | 3 | -0.75 | 0.2131 | 0.2101 | 0.0016 | 0 | 2 | 1 | 1 | 0.25 | 2.0317 | 2.0370 | 0.0030 |
| 0 | -2 | 3 | 3 | -0.75 | 0.7963 | 0.7951 | 0.0032 | 2 | 2 | 1 | 1 | 0.25 | 2.4890 | 2.4942 | 0.0028 |
| 2 | -2 | 3 | 3 | -0.75 | 2.1912 | 2.1874 | 0.0047 | -2 | -2 | 3 | 1 | 0.25 | 0.1130 | 0.1115 | 0.0012 |
| -2 | 0 | 3 | 3 | -0.75 | 0.7963 | 0.7987 | 0.0032 | 0 | -2 | 3 | 1 | 0.25 | 0.6937 | 0.6996 | 0.0032 |
| 0 | 0 | 3 | 3 | -0.75 | 1.3373 | 1.3401 | 0.0034 | 2 | -2 | 3 | 1 | 0.25 | 2.1070 | 2.1105 | 0.0049 |
| 2 | 0 | 3 | 3 | -0.75 | 2.5326 | 2.5286 | 0.0041 | -2 | 0 | 3 | 1 | 0.25 | 0.4602 | 0.4592 | 0.0020 |
| -2 | 2 | 3 | 3 | -0.75 | 2.1912 | 2.1938 | 0.0047 | 0 | 0 | 3 | 1 | 0.25 | 0.8981 | 0.8967 | 0.0031 |
| 0 | 2 | 3 | 3 | -0.75 | 2.5326 | 2.5335 | 0.0041 | 2 | 0 | 3 | 1 | 0.25 | 2.1598 | 2.1610 | 0.0048 |
| 2 | 2 | 3 | 3 | -0.75 | 3.2927 | 3.2937 | 0.0036 | -2 | 2 | 3 | 1 | 0.25 | 2.0154 | 2.0210 | 0.0031 |
| -2 | -2 | 1 | 1 | -0.50 | 0.0170 | 0.0171 | 0.0003 | 0 | 2 | 3 | 1 | 0.25 | 2.1203 | 2.1202 | 0.0031 |
| 0 | -2 | 1 | 1 | -0.50 | 0.4071 | 0.4061 | 0.0018 | 2 | 2 | 3 | 1 | 0.25 | 2.7083 | 2.7092 | 0.0039 |
| 2 | -2 | 1 | 1 | -0.50 | 2.0116 | 2.0141 | 0.0031 | -2 | -2 | 1 | 3 | 0.25 | 0.1130 | 0.1135 | 0.0012 |
| -2 | 0 | 1 | 1 | -0.50 | 0.4071 | 0.4100 | 0.0018 | 0 | -2 | 1 | 3 | 0.25 | 0.4602 | 0.4575 | 0.0020 |
| 0 | 0 | 1 | 1 | -0.50 | 0.7444 | 0.7427 | 0.0020 | 2 | -2 | 1 | 3 | 0.25 | 2.0154 | 2.0163 | 0.0031 |
| 2 | 0 | 1 | 1 | -0.50 | 2.1069 | 2.1095 | 0.0028 | -2 | 0 | 1 | 3 | 0.25 | 0.6937 | 0.6964 | 0.0032 |
| -2 | 2 | 1 | 1 | -0.50 | 2.0116 | 2.0132 | 0.0031 | 0 | 0 | 1 | 3 | 0.25 | 0.8981 | 0.8966 | 0.0031 |
| 0 | 2 | 1 | 1 | -0.50 | 2.1069 | 2.1059 | 0.0028 | 2 | 0 | 1 | 3 | 0.25 | 2.1203 | 2.1242 | 0.0031 |
| 2 | 2 | 1 | 1 | -0.50 | 2.6910 | 2.6933 | 0.0023 | -2 | 2 | 1 | 3 | 0.25 | 2.1070 | 2.1111 | 0.0049 |
| -2 | -2 | 3 | 1 | -0.50 | 0.1150 | 0.1161 | 0.0012 | 0 | 2 | 1 | 3 | 0.25 | 2.1598 | 2.1604 | 0.0048 |
| 0 | -2 | 3 | 1 | -0.50 | 0.6991 | 0.7005 | 0.0032 | 2 | 2 | 1 | 3 | 0.25 | 2.7083 | 2.7026 | 0.0038 |
| 2 | -2 | 3 | 1 | -0.50 | 2.1121 | 2.1105 | 0.0049 | -2 | -2 | 3 | 3 | 0.25 | 0.1978 | 0.1970 | 0.0016 |
| -2 | 0 | 3 | 1 | -0.50 | 0.4992 | 0.4996 | 0.0020 | 0 | -2 | 3 | 3 | 0.25 | 0.7410 | 0.7399 | 0.0032 |
| 0 | 0 | 3 | 1 | -0.50 | 1.0225 | 1.0214 | 0.0030 | 2 | -2 | 3 | 3 | 0.25 | 2.1187 | 2.1201 | 0.0049 |
| 2 | 0 | 3 | 1 | -0.50 | 2.2766 | 2.2691 | 0.0044 | -2 | 0 | 3 | 3 | 0.25 | 0.7410 | 0.7375 | 0.0032 |
| -2 | 2 | 3 | 1 | -0.50 | 2.0499 | 2.0538 | 0.0030 | 0 | 0 | 3 | 3 | 0.25 | 1.1141 | 1.1066 | 0.0037 |
| 0 | 2 | 3 | 1 | -0.50 | 2.2707 | 2.2755 | 0.0028 | 2 | 0 | 3 | 3 | 0.25 | 2.2534 | 2.2634 | 0.0047 |
| 2 | 2 | 3 | 1 | -0.50 | 2.9551 | 2.9588 | 0.0033 | -2 | 2 | 3 | 3 | 0.25 | 2.1187 | 2.1160 | 0.0049 |
| -2 | -2 | 1 | 3 | -0.50 | 0.1150 | 0.1154 | 0.0012 | 0 | 2 | 3 | 3 | 0.25 | 2.2534 | 2.2474 | 0.0047 |
| 0 | -2 | 1 | 3 | -0.50 | 0.4992 | 0.5011 | 0.0020 | 2 | 2 | 3 | 3 | 0.25 | 2.8616 | 2.8564 | 0.0047 |
| 2 | -2 | 1 | 3 | -0.50 | 2.0499 | 2.0513 | 0.0030 | -2 | -2 | 1 | 1 | 0.50 | 0.0159 | 0.0157 | 0.0003 |
| -2 | 0 | 1 | 3 | -0.50 | 0.6991 | 0.6995 | 0.0032 | 0 | -2 | 1 | 1 | 0.50 | 0.4001 | 0.3979 | 0.0018 |
| 0 | 0 | 1 | 3 | -0.50 | 1.0225 | 1.0284 | 0.0030 | 2 | -2 | 1 | 1 | 0.50 | 2.0085 | 2.0071 | 0.0031 |
| 2 | 0 | 1 | 3 | -0.50 | 2.2707 | 2.2709 | 0.0028 | -2 | 0 | 1 | 1 | 0.50 | 0.4001 | 0.3998 | 0.0018 |
| -2 | 2 | 1 | 3 | -0.50 | 2.1121 | 2.1030 | 0.0049 | 0 | 0 | 1 | 1 | 0.50 | 0.5984 | 0.5951 | 0.0021 |
| 0 | 2 | 1 | 3 | -0.50 | 2.2766 | 2.2712 | 0.0044 | 2 | 0 | 1 | 1 | 0.50 | 2.0159 | 2.0151 | 0.0031 |
| 2 | 2 | 1 | 3 | -0.50 | 2.9551 | 2.9544 | 0.0033 | -2 | 2 | 1 | 1 | 0.50 | 2.0085 | 2.0097 | 0.0031 |
| -2 | -2 | 3 | 3 | -0.50 | 0.2127 | 0.2136 | 0.0017 | 0 | 2 | 1 | 1 | 0.50 | 2.0159 | 2.0109 | 0.0031 |
| 0 | -2 | 3 | 3 | -0.50 | 0.7893 | 0.7910 | 0.0032 | 2 | 2 | 1 | 1 | 0.50 | 2.4001 | 2.4015 | 0.0029 |
| 2 | -2 | 3 | 3 | -0.50 | 2.1701 | 2.1558 | 0.0047 | -2 | -2 | 3 | 1 | 0.50 | 0.1108 | 0.1125 | 0.0012 |
| -2 | 0 | 3 | 3 | -0.50 | 0.7893 | 0.7861 | 0.0032 | 0 | -2 | 3 | 1 | 0.50 | 0.6918 | 0.6950 | 0.0032 |
| 0 | 0 | 3 | 3 | -0.50 | 1.2894 | 1.2883 | 0.0035 | 2 | -2 | 3 | 1 | 0.50 | 2.1066 | 2.1122 | 0.0049 |
| 2 | 0 | 3 | 3 | -0.50 | 2.4616 | 2.4580 | 0.0043 | -2 | 0 | 3 | 1 | 0.50 | 0.4404 | 0.4393 | 0.0020 |
| -2 | 2 | 3 | 3 | -0.50 | 2.1701 | 2.1750 | 0.0047 | 0 | 0 | 3 | 1 | 0.50 | 0.8454 | 0.8463 | 0.0031 |
| 0 | 2 | 3 | 3 | -0.50 | 2.4616 | 2.4605 | 0.0043 | 2 | 0 | 3 | 1 | 0.50 | 2.1297 | 2.1201 | 0.0048 |
| 2 | 2 | 3 | 3 | -0.50 | 3.1973 | 3.1990 | 0.0040 | -2 | 2 | 3 | 1 | 0.50 | 2.0103 | 2.0068 | 0.0031 |
| -2 | -2 | 1 | 1 | -0.25 | 0.0170 | 0.0173 | 0.0003 | 0 | 2 | 3 | 1 | 0.50 | 2.0722 | 2.0671 | 0.0032 |
| 0 | -2 | 1 | 1 | -0.25 | 0.4060 | 0.4071 | 0.0019 | 2 | 2 | 3 | 1 | 0.50 | 2.6050 | 2.6047 | 0.0040 |
| 2 | -2 | 1 | 1 | -0.25 | 2.0099 | 2.0067 | 0.0031 | -2 | -2 | 1 | 3 | 0.50 | 0.1108 | 0.1114 | 0.0012 |
| -2 | 0 | 1 | 1 | -0.25 | 0.4060 | 0.4043 | 0.0018 | 0 | -2 | 1 | 3 | 0.50 | 0.4404 | 0.4409 | 0.0020 |
| 0 | 0 | 1 | 1 | -0.25 | 0.7143 | 0.7141 | 0.0021 | 2 | -2 | 1 | 3 | 0.50 | 2.0103 | 2.0090 | 0.0031 |
| 2 | 0 | 1 | 1 | -0.25 | 2.0789 | 2.0817 | 0.0029 | -2 | 0 | 1 | 3 | 0.50 | 0.6918 | 0.6916 | 0.0032 |
| -2 | 2 | 1 | 1 | -0.25 | 2.0099 | 2.0042 | 0.0031 | 0 | 0 | 1 | 3 | 0.50 | 0.8454 | 0.8437 | 0.0032 |
| 0 | 2 | 1 | 1 | -0.25 | 2.0789 | 2.0805 | 0.0029 | 2 | 0 | 1 | 3 | 0.50 | 2.0722 | 2.0655 | 0.0032 |
| 2 | 2 | 1 | 1 | -0.25 | 2.6308 | 2.6294 | 0.0025 | -2 | 2 | 1 | 3 | 0.50 | 2.1066 | 2.1072 | 0.0049 |
| -2 | -2 | 3 | 1 | -0.25 | 0.1149 | 0.1156 | 0.0012 | 0 | 2 | 1 | 3 | 0.50 | 2.1297 | 2.1333 | 0.0048 |
| 0 | -2 | 3 | 1 | -0.25 | 0.6978 | 0.6982 | 0.0032 | 2 | 2 | 1 | 3 | 0.50 | 2.6050 | 2.6071 | 0.0040 |
| 2 | -2 | 3 | 1 | -0.25 | 2.1098 | 2.1150 | 0.0049 | -2 | -2 | 3 | 3 | 0.50 | 0.1850 | 0.1857 | 0.0016 |
| -2 | 0 | 3 | 1 | -0.25 | 0.4897 | 0.4864 | 0.0020 | 0 | -2 | 3 | 3 | 0.50 | 0.7190 | 0.7179 | 0.0032 |
| 0 | 0 | 3 | 1 | -0.25 | 0.9850 | 0.9844 | 0.0031 | 2 | -2 | 3 | 3 | 0.50 | 2.1096 | 2.1138 | 0.0049 |
| 2 | 0 | 3 | 1 | -0.25 | 2.2347 | 2.2283 | 0.0045 | -2 | 0 | 3 | 3 | 0.50 | 0.7190 | 0.7188 | 0.0032 |
| -2 | 2 | 3 | 1 | -0.25 | 2.0357 | 2.0390 | 0.0030 | 0 | 0 | 3 | 3 | 0.50 | 1.0365 | 1.0370 | 0.0037 |
| 0 | 2 | 3 | 1 | -0.25 | 2.2205 | 2.2150 | 0.0030 | 2 | 0 | 3 | 3 | 0.50 | 2.1850 | 2.1887 | 0.0048 |
| 2 | 2 | 3 | 1 | -0.25 | 2.8802 | 2.8789 | 0.0035 | -2 | 2 | 3 | 3 | 0.50 | 2.1096 | 2.1060 | 0.0049 |
| -2 | -2 | 1 | 3 | -0.25 | 0.1149 | 0.1143 | 0.0012 | 0 | 2 | 3 | 3 | 0.50 | 2.1850 | 2.1771 | 0.0048 |
| 0 | -2 | 1 | 3 | -0.25 | 0.4897 | 0.4880 | 0.0020 | 2 | 2 | 3 | 3 | 0.50 | 2.7190 | 2.7211 | 0.0048 |
| 2 | -2 | 1 | 3 | -0.25 | 2.0357 | 2.0285 | 0.0030 | -2 | -2 | 1 | 1 | 0.75 | 0.0143 | 0.0142 | 0.0003 |
| -2 | 0 | 1 | 3 | -0.25 | 0.6978 | 0.6983 | 0.0032 | 0 | -2 | 1 | 1 | 0.75 | 0.3990 | 0.3968 | 0.0018 |
| 0 | 0 | 1 | 3 | -0.25 | 0.9850 | 0.9894 | 0.0031 | 2 | -2 | 1 | 1 | 0.75 | 2.0085 | 2.0130 | 0.0031 |
| 2 | 0 | 1 | 3 | -0.25 | 2.2205 | 2.2216 | 0.0029 | -2 | 0 | 1 | 1 | 0.75 | 0.3990 | 0.3984 | 0.0018 |
| -2 | 2 | 1 | 3 | -0.25 | 2.1098 | 2.1119 | 0.0049 | 0 | 0 | 1 | 1 | 0.75 | 0.5400 | 0.5404 | 0.0021 |
| 0 | 2 | 1 | 3 | -0.25 | 2.2347 | 2.2337 | 0.0045 | 2 | 0 | 1 | 1 | 0.75 | 2.0089 | 2.0021 | 0.0031 |
| 2 | 2 | 1 | 3 | -0.25 | 2.8802 | 2.8847 | 0.0035 | -2 | 2 | 1 | 1 | 0.75 | 2.0085 | 2.0073 | 0.0031 |
| -2 | -2 | 3 | 3 | -0.25 | 0.2106 | 0.2091 | 0.0016 | 0 | 2 | 1 | 1 | 0.75 | 2.0089 | 2.0071 | 0.0031 |
| 0 | -2 | 3 | 3 | -0.25 | 0.7770 | 0.7755 | 0.0032 | 2 | 2 | 1 | 1 | 0.75 | 2.2848 | 2.2821 | 0.0030 |
| 2 | -2 | 3 | 3 | -0.25 | 2.1504 | 2.1470 | 0.0048 | -2 | -2 | 3 | 1 | 0.75 | 0.1080 | 0.1079 | 0.0012 |
| -2 | 0 | 3 | 3 | -0.25 | 0.7770 | 0.7775 | 0.0032 | 0 | -2 | 3 | 1 | 0.75 | 0.6910 | 0.6884 | 0.0032 |
| 0 | 0 | 3 | 3 | -0.25 | 1.2373 | 1.2316 | 0.0036 | 2 | -2 | 3 | 1 | 0.75 | 2.1066 | 2.1083 | 0.0049 |
| 2 | 0 | 3 | 3 | -0.25 | 2.3922 | 2.3860 | 0.0045 | -2 | 0 | 3 | 1 | 0.75 | 0.4175 | 0.4212 | 0.0020 |
| -2 | 2 | 3 | 3 | -0.25 | 2.1504 | 2.1499 | 0.0048 | 0 | 0 | 3 | 1 | 0.75 | 0.7811 | 0.7780 | 0.0032 |
| 0 | 2 | 3 | 3 | -0.25 | 2.3922 | 2.3901 | 0.0045 | 2 | 0 | 3 | 1 | 0.75 | 2.1102 | 2.1084 | 0.0049 |
| 2 | 2 | 3 | 3 | -0.25 | 3.0950 | 3.0949 | 0.0042 | -2 | 2 | 3 | 1 | 0.75 | 2.0086 | 2.0044 | 0.0031 |
| -2 | -2 | 1 | 1 | 0.00 | 0.0169 | 0.0170 | 0.0003 | 0 | 2 | 3 | 1 | 0.75 | 2.0307 | 2.0336 | 0.0032 |
| 0 | -2 | 1 | 1 | 0.00 | 0.4042 | 0.4020 | 0.0018 | 2 | 2 | 3 | 1 | 0.75 | 2.4794 | 2.4821 | 0.0042 |
| 2 | -2 | 1 | 1 | 0.00 | 2.0090 | 2.0062 | 0.0031 | -2 | -2 | 1 | 3 | 0.75 | 0.1080 | 0.1064 | 0.0012 |
| -2 | 0 | 1 | 1 | 0.00 | 0.4042 | 0.4036 | 0.0018 | 0 | -2 | 1 | 3 | 0.75 | 0.4175 | 0.4144 | 0.0019 |
| 0 | 0 | 1 | 1 | 0.00 | 0.6810 | 0.6828 | 0.0021 | 2 | -2 | 1 | 3 | 0.75 | 2.0086 | 2.0102 | 0.0031 |
| 2 | 0 | 1 | 1 | 0.00 | 2.0534 | 2.0539 | 0.0030 | -2 | 0 | 1 | 3 | 0.75 | 0.6910 | 0.6919 | 0.0032 |
| -2 | 2 | 1 | 1 | 0.00 | 2.0090 | 2.0085 | 0.0031 | 0 | 0 | 1 | 3 | 0.75 | 0.7811 | 0.7861 | 0.0032 |
| 0 | 2 | 1 | 1 | 0.00 | 2.0534 | 2.0509 | 0.0030 | 2 | 0 | 1 | 3 | 0.75 | 2.0307 | 2.0289 | 0.0032 |
| 2 | 2 | 1 | 1 | 0.00 | 2.5643 | 2.5646 | 0.0026 | -2 | 2 | 1 | 3 | 0.75 | 2.1066 | 2.1011 | 0.0049 |
| -2 | -2 | 3 | 1 | 0.00 | 0.1143 | 0.1134 | 0.0012 | 0 | 2 | 1 | 3 | 0.75 | 2.1102 | 2.1144 | 0.0049 |
| 0 | -2 | 3 | 1 | 0.00 | 0.6959 | 0.6977 | 0.0032 | 2 | 2 | 1 | 3 | 0.75 | 2.4794 | 2.4844 | 0.0042 |
| 2 | -2 | 3 | 1 | 0.00 | 2.1080 | 2.1155 | 0.0049 | -2 | -2 | 3 | 3 | 0.75 | 0.1647 | 0.1647 | 0.0015 |
| -2 | 0 | 3 | 1 | 0.00 | 0.4766 | 0.4760 | 0.0020 | 0 | -2 | 3 | 3 | 0.75 | 0.6982 | 0.6975 | 0.0032 |
| 0 | 0 | 3 | 1 | 0.00 | 0.9439 | 0.9451 | 0.0031 | 2 | -2 | 3 | 3 | 0.75 | 2.1066 | 2.1056 | 0.0049 |
| 2 | 0 | 3 | 1 | 0.00 | 2.1955 | 2.2002 | 0.0047 | -2 | 0 | 3 | 3 | 0.75 | 0.6982 | 0.6971 | 0.0032 |
| -2 | 2 | 3 | 1 | 0.00 | 2.0240 | 2.0166 | 0.0031 | 0 | 0 | 3 | 3 | 0.75 | 0.9353 | 0.9313 | 0.0036 |
| 0 | 2 | 3 | 3 | 0.75 | 2.1257 | 2.1320 | 0.0049 | 2 | 0 | 3 | 3 | 0.75 | 2.1257 | 2.1249 | 0.0049 |
| 2 | 2 | 3 | 3 | 0.75 | 2.5370 | 2.5361 | 0.0050 | -2 | 2 | 3 | 3 | 0.75 | 2.1066 | 2.1121 | 0.0049 |

**References**

1 Nadarajah S, Kotz S. Exact Distribution of the Max/Min of Two Gaussian Random Variables. *IEEE Transactions on Very Large Scale Integration (VLSI) Systems* 2008;**16**:210–2. doi:10.1109/TVLSI.2007.912191

2 Owen DB. A table of normal integrals. *Communications in Statistics - Simulation and Computation* 1980;**9**:389–419. doi:10.1080/03610918008812164
